# Medical Opinion and Movement

**Published:** 1908-12-12

**Authors:** 


### Medical Opinion and Movement.

Although the delivery of a living and viable child ectopically conceived is no longer, thanks to the progress of operative gynæcology, so extremely rare an event as it used to be, still the personal experience of Dr. Werder, which includes three cases of this nature, is most exceptional. The three cases are recorded in the *American Journal of Obstetrics*. Two of the mothers recovered satisfactorily, but the other died a few hours after operation from the shock of profuse hæmorrhage: of the babies, none survived, but one lived for three days, the other two only a few hours each. Deformities of the legs and arms were noticed in all three children, and of the face in one. In each case it seems probable that the tubal rupture was primary only, and in one it is thought that the original amniotic sac persisted throughout, though it contained but two ounces of fluid. The placentæ were all very large: two were adherent even to loops of intestine. The author, when considering the time of election for operation in cases of ectopic gestation which have already gone more than half way to term, and in which the fœtus is living, expresses himself in favour of postponing interference until as near term as possible. The danger of secondary rupture at this stage is very slight, and although the child has but an ill chance of life compared with those conceived *in utero*, such chance as it has is vastly greater at nine months than at seven or eight. He quotes a series of 122 such infants collected by Sittern, whereof 63 survived the first month. As regards the old difficulty of dealing with the placenta, Dr. Werder is in favour of complete removal at the time of delivery: he believes neither in postponing laparotomy for two months after the death of the fœtus, nor in draining the sac through the abdominal wound, nor in closing the abdomen and leaving the placenta *in situ*. He thinks the ligature of the ovarian arteries is one of the most important features of the operation; and, if these cannot be secured, would temporarily occlude the aorta by Halstead's clamp.

At the first meeting of the Section of the Royal Society of Medicine in which is now merged the old Society for the Study of Disease in Children, Mr. Sydney Stephenson made some interesting remarks, *à propos* of congenital morbus cordis, upon retinal changes in that disease. The retinal veins may be dilated to three or four times the normal width and filled with dark-coloured blood; the arteries may also be affected, and the optic discs are frequently hazy. Mr. Stephenson feels inclined to distinguish two kinds of retinal changes: one in which the stress falls on the veins alone, and the other in which both veins and arteries are involved. In the latter case he thinks it possible that a mixing of arterial and venous blood has taken place, as by imperfection of the septum ventriculorum or patent ductus arteriosus, and also that an absence of arterial changes in the retina is to some extent evidence of some other kind of congenital heart lesion. In one case of a child admitted comatose to the Evelina Hospital the diagnosis was made practically on the findings by the ophthalmoscope. In the subsequent discussion the President of the Section (Dr. George Carpenter) narrated a case of his own many years ago, in which the arteries and veins of the retina were the largest and most tortuous he had ever seen. After death he made microscopical examinations of many of the organs and found tortuous, thick, and fibrillated capillaries everywhere except in the skin.

Maternal impressions and their supposed transmission to the fœtus as blemishes or deformities constitute one of those apparently simple problems which are much more intricate than they appear to be. It is at first sight merely a question of evidence as to whether such prenatal influences do affect the unborn child; but the evidence requires an extraordinary amount of sifting, and it further requires interpretation by a competent embryologist. Dr. Melland reports in the *British Journal of Children's Diseases* an apparent case of clearly established maternal impression such as would pass for proof were it not revised by the light of embryological researches. A woman during the last three months of her pregnancy came into daily contact at her work with a boy of twelve who possessed an accessory tragus to his right ear. She was very much struck by this deformity, the like of which she had never seen or heard of before. The boy, it should be added, was unconnected by blood with the prospective mother. When her child was born it was found to have an accessory tragus almost exactly similar to that of the boy, but on the left ear. This abnormality she attributed to the impression received by her from the boy: and, as Dr. Melland says, it seems not an unreasonable idea. But the fatal flaw, he proceeds, comes in when it is remembered that the woman had never seen, or at any rate never noticed, the boy during the first six months of gestation; whereas the external ear acquires its adult form as early as the sixth week. The author goes further, for he expresses agreement with those who totally deny the possibility of maternal impression often, or ever, causing a defect in the fœtus closely resembling the thing producing the impression.

Dr. Fritz Lesser, reporting on the sero-reaction of Wassermann for syphilis, asserts that the reaction is always positive in general paralysis, whereas in patients affected with tertiary symptoms of the skin it is positive only in 82.6 per cent. In cerebral gumma the sero-reaction is again frequently negative, so that a negative reaction is of no service in the differential diagnosis of syphilis from cerebral tumour. The explanation of these facts is not at all obvious. Blaschko is of opinion that about one-third of all syphilitics terminate in locomotor ataxia, general paralysis, or aneurysm, and reasoning from the fact that cases of general paralysis always show a positive reaction, he suggests that every syphilitic who shows a negative reaction in the later years of the disease is in no danger of being among that number. On the other hand all syphilitics who present the reaction of Wassermann should be under treatment until the reaction becomes negative. This would constitute a strong prophylactic measure against the development of locomotor ataxia or general paralysis. The duration of the treatment necessary to obtain the disappearance of Wassermann's reaction varies in different individuals. Iodide of potassium is said to effect the end in view. There is undoubtedly much yet to learn in regard to the bacteriology of the disease, but it seems not improbable that at no distant date by this reaction or some other of a similar nature it may be possible to gauge accurately the effect of treatment on the course of the disease, and determine exactly its eradication from the system.

---

THE subcutaneous injection of paraffin is now a well-recognised surgical procedure for the purpose of remedying certain deformities. It has also apparently been found a useful means of simulating disease. Dr. Violine reports two cases in which paraffin injections were resorted to in order to obtain freedom from military service in Russia. The first case was that of a young soldier who complained of swellings in the leg, and on examination several large tumours were found underlying the skin and situated in the soft parts of the leg. They were cartilaginous in consistency, and it was supposed represented a rare form of myositis ossificans. A little while after, however, another recruit presented himself with exactly the same kind of tumours. The coincidence aroused suspicion, and the new patient was placed under observation. Manipulation of the tumours showed that they were painless even on hard pressure, and it was found that they changed in shape by manipulation, as if they were formed of wax or paraffin. Red points were also detected, suggesting the pricks of an injection needle. Suspecting the nature of these tumours it was proposed to the patient that they should be excised, but this was resolutely refused by him. In order, therefore, to verify the diagnosis hot plates of zinc were placed against these swellings, and in each case at the end of a few minutes they changed shape and became flattened. Similar cases have also been reported by other German and Russian surgeons, in which elephantiasis of the scrotum and large exostoses of the forearm have been simulated.

---

SCHISTOSOMIASIS Japonica, which was discovered in Japan, appears to be endemic in that country, and is also found in China and in the Philippine Islands. For some years Japanese physicians have known of an affection characterised by enlargement of the liver and spleen, with diarrhœa, anæmia, and ascites. In 1887 Majima discovered the eggs of an unknown parasite in the liver, and this was confirmed by subsequent observers. In 1903 Kasai found the same eggs in the fæces of patients suffering from the disease, and in 1904 Katsurada discovered the parasite itself in the blood from the portal veins of cats coming from an affected district, as well as the eggs in the fæces of the same cats. The parasite appeared similar to the Bilharzia hæmatobia. On further study of this parasite by Tsuchiya and others it is found that this parasite differs in many respects from the Bilharzia hæmatobia in regard to size, shape, and organs, as well as in the disease which it sets up. The life history of this parasite and the course of the disease appear to be as follows. The parasite is taken into the system with impure water, and accordingly the disease is more common in the hot weather, occurs more frequently in males than females, and especially attacks children of the poorer classes. The eggs are deposited in the walls of the intestinal tract, and are carried thence into the portal circulation to the liver and spleen, giving rise to enlargement of these organs. The development of the disease is said to be very insidious so as to easily escape diagnosis in the early stages. Later the hypertrophy of the liver becomes considerable and is accompanied by epigastric pain, diarrhœa, dyspnœa, epistaxis, and later by ascites. The disease may suddenly assume a severe form, but usually the patient retains his normal capacity for work for a period of 20 or 30 years.

---

THE treatment by superheated air forms the subject of an interesting article by Dr. H. Dausset in *Le Progrès Médical*. He has carried out this form of treatment on an extensive scale, and he passes in review the many affections from which he has been able to obtain relief by this means. Considerable skill and practical knowledge are necessary for the successful application of the method, as may be judged from the wide range of temperature employed under different conditions. As a bath superheated air is used at temperatures of 50° to 120° C., as a douche from 150° to 300° C., and as a cautery from 300° to 500° C. Dr. Dausset admits that much greater experience is required in order to obtain the precise indications and conditions for the treatment. One of the most important properties of the "ærothermotherapy" consists in its analgesic power. It is therefore especially indicated in all neuralgic conditions of a rheumatic nature or arising from cold. Sciatica, lumbago, and intercostal neuralgia have yielded to the treatment. Schultz obtained 12 cures in 20 cases of tic douloureux after all other known forms of treatment had failed. The hyperæmic effect of hot air renders it useful in a number of affections of the bones and joints, especially of a rheumatic or traumatic character. The hot-air bath may be used in these cases or the douche combined with massage. It regulates the circulation, increases the rapidity of absorption of the inflammatory products, and relieves the pain so as to allow freer movement of the articular surfaces. Hot-air applications have also been used successfully for all kinds of skin affections, dermatitis, eczema, various ulcers, diabetic gangrene, and as a cautery for the removal of lupus and superficial epitheliomata. Dr. Dausset gives a yet further list of morbid conditions in which hot air in some form has been found of value, so that its range of possible therapeutic use appears to be very wide. The apparatus and the technical skill required are, however, serious deterrents to its more general adoption by the profession.

It was at a meeting in Berlin some ten years ago that Kustner somewhat startled the medical world by openly advocating the practice of allowing newly-delivered women to leave their beds within a few hours of delivery. As far as this country is concerned, it cannot be said that his teaching has been adopted by any accoucheurs of note; but in Germany it would seem that he is gradually making converts, for a recent number of the *Centralblatt für Gynækologie* contains the reports of a number of cases treated by Dr. Alvens-Leben according to Kustner's method at the clinique of Kiel. At first, early rising was only allowed to primiparæ who had had a normal labour, but the results were so favourable that later on multiparæ were allowed similar license, even when labour had been terminated by an obstetrical operation such as forceps, version, etc. At a later date hæmorrhage was not considered a contra-indication, the old system of lying-up only being enforced in cases of very prolonged labour with much damage to the soft parts or of extensive lacerations of the perinæum. Robust patients are made to rise the day after delivery; the delicate are kept in bed for three days. In no case, however, is a patient forced to get up should she feel too weak to do so. The ordinary treatment of the breasts and vulva is carried out in all cases. Carefully graduated exercises are imposed on the patient. The first exercise consists in voluntarily contracting the abdominal muscles as forcibly as possible a dozen times or so. This is repeated four or five times in the course of the day. Then the patient, lying on her back, is made to rise into the sitting posture, at first with help, later without; the patient, still in the dorsal decubitus, is next made to rotate her extended legs. As soon as the patient has got up she is made to extend, flex, and rotate the trunk.

---

All these exercises are given under supervision so as to obviate any exaggerated movements. On first getting up the patient is allowed to sit for an hour on a couch, and, after taking two or three steps round this, is put back to bed. Next day she is allowed up twice, and walks a little farther. During these exercises she wears a tight binder and a vulval pad. In ninety cases the progress of the patients was absolutely normal. Six patients had to be sent back to bed owing to weakness, vertigo, irregular pulse, etc. In ten cases fever was present, due to purulent lochia, mastitis, etc., but was temporary, and quickly passed away. Involution of the uterus took place rapidly. The lochia, though at first abundant, at once decreased, and soon stopped altogether. The genitalia and abdominal muscles quickly resumed their normal condition. Strength and appetite quickly returned, and the patients benefited in every way by the relief from the monotony of lying-in. It must be understood, however, that by early rising the author does not mean early resumption of household occupations. He is of opinion that unless the medical attendant is able to supervise strictly the patient's exercises, it is better to have recourse to the old treatment of lying in bed for ten days or so.

A writer in the *Medical Record* gives an interesting account of the methods of the Mexican midwife. The patient was a Peon, or working-class woman, of nearly pure Indian descent, and "born and raised in dirt." There were two midwives in attendance, one being stationed behind, holding the patient's hips between her knees, both arms about the waist just above the uterus, a sort of human chair. As each pain came on she hugged with all her force, which was considerable. Hanging in front of the patient was a rope of old clothes, bags, and towels, up which the patient drew herself when the pains began. The chief midwife was in front. She was squatted on her heels, with a hand on either of the patient's hips under the skirt. As each pain began the patient started climbing the rope, dragging the posterior midwife up with her, until they were nearly erect, the patient resting on the toes, the knees being flexed and widely separated. After one long pain the child slipped into the hands of the midwife; the woman was allowed to slip to the floor while the cord was being tied and cut. Without moving the child from between the mother's thighs, they made her climb up the rope once more, and after a couple of shakes and a gentle pull on the cord the membranes came away. The grandmother brought a good, generous bit of lard on the ends of the fingers, which was transferred to the midwife, who daubed it on the stump of the cord, and the third stage was completed. All with one accord lighted cigarettes, the grandmother bringing a lighted one for the patient. There was no laceration of the perinæum, and the patient did excellently!

---

Tabora in the *Munch. Med. Woch.* discusses the use of atropine in the treatment of certain cases of gastric ulcer. It is indicated especially in cases in which other medical treatment has failed, and the success of surgical interference seems doubtful. It should not be given in uncomplicated cases of gastric ulceration. When there is hyperchlorhydria and motor insufficiency, atropine diminishes the secretion of hydrochloric acid, relieves pain, and prevents spasmodic contraction of the stomach. The drug is, moreover, a mild narcotic. There are, however, two obvious disadvantages—a loss of accommodation necessitating the use of glasses, and dryness of the mouth and throat. An overdose aggravates rather than relieves the sensibility of the secretory nerves. The patient is first of all put to bed fasting for two or three days, and merely receives water injections by the rectum. Then milk is commenced in teaspoonful doses, which are gradually increased in number and cream added. At this point one milligram of atropine is injected *sub cutem* twice daily. The diet is gradually increased by the addition of broths, eggs, etc., until after two months finely minced meat is being taken. The atropine injections are continued for about six to ten weeks only. Symptoms cease almost from the commencement of treatment and in many cases gastric hypersecretion is completely arrested. The motor function of the stomach is at the same time greatly improved. The author believes that this method is superior to all others in ensuring complete rest for the stomach.

